# COVID-19 incidence among Kenyan patients with and without inflammatory rheumatic disease

**DOI:** 10.4102/jphia.v16i1.1409

**Published:** 2025-10-31

**Authors:** Benwillies Onchong’a, Tuulikki Sokka-Isler, Pekka Mäntyselkä, Ari Voutilainen

**Affiliations:** 1 Institute of Public Health and Clinical Nutrition, Faculty of Health Sciences, University of Eastern Finland, Kuopio, Finland; 2 Department of Rheumatic Diseases, Jasmota Hospital, Nairobi, Kenya; 3 Institute of Clinical Medicine, Faculty of Health Sciences, University of Eastern Finland, Kuopio, Finland; 4 Wellbeing Services County of Central Finland, Hospital Nova, Jyväskylä, Finland; 5 Teaching Clinic, Wellbeing Services County of North Savo, Kuopio, Finland

**Keywords:** COVID-19, comorbidity, hazard ratio, inflammatory rheumatic diseases, risk factors, incidence, Kenya

## Abstract

**Background:**

Inflammatory rheumatic diseases (IRDs) have been considered potential risk factors for COVID-19, but evidence from Africa remains limited.

**Aim:**

To investigate the association between IRDs and COVID-19 among general patients after hospital discharge in Nairobi, Kenya.

**Setting:**

The prospective cohort study was conducted at Mbagathi County Hospital, a major public hospital in Nairobi, Kenya.

**Methods:**

Patients were classified as IRD and non-IRD cases based on admission diagnosis. After discharge, 348 IRD and 2951 non-IRD patients were followed up for 2 years or until death. Cox proportional hazard models adjusted for baseline characteristics were executed to predict COVID-19 hazard in patients with versus without IRDs.

**Results:**

The cohort included 46.2% women. IRD patients were older (mean 64 years vs. 62 years; *p* < 0.001), more frequently alcohol drinkers (17.0% vs. 9.5%; *p* < 0.001), less often vaccinated against COVID-19 (74.0% vs. 78.0%; *p* = 0.031) and had higher body mass index (BMI) (mean 26.3 kg/m^2^ vs. 25.3 kg/m^2^; *p* < 0.001). The 2-year COVID-19 incidence rate per 100 person-years was 4.5 (95% confidence interval [CI]: 3–7) in IRD patients and 5.0 (95% CI: 4–6) in non-IRD patients. The age- and sex-adjusted hazard of COVID-19 among IRD versus non-IRD patients was 0.9 (95% CI: 0.6–1.4; *p* = 0.667).

**Conclusion:**

Inflammatory rheumatic diseases did not increase COVID-19 risk in this Kenyan cohort.

**Contribution:**

This study provides valuable African data on IRDs and COVID-19 risk, reflecting potential regional features in clinical practice and public health strategies.

## Introduction

Since its outbreak in 2019, novel COVID-19 has had considerable global health effects on patient populations. Older age and the presence of comorbidities have largely been reported to increase the risk for severe disease.^1^ Among patient groups vulnerable to severe COVID-19 outcomes, inflammatory rheumatic diseases (IRDs) have been of concern because of an exaggerated inflammatory reaction associated with IRDs.^2^ A higher COVID-19 infection rate among IRD patients has been associated with an immune dysregulation caused by IRDs,^3^ which results in a poor immune response against viral replication.^4^

Many population-based studies from high-resource settings have reported an increased risk of COVID-19 infection among IRD patients,^2,5,6^ although contrary findings also exist.^7^ These conflicting results may reflect differences in treatment protocols, healthcare systems or population characteristics. For instance, treatments used for IRDs, such as hydroxychloroquine and baricitinib, may have antiviral effects that could potentially weaken COVID-19 impact.^8^ A review conducted on studies of COVID-19 incidence and outcomes in autoinflammatory disease cohorts reported that even though patients with IRD receiving biologic disease-modifying antirheumatic drugs known as DMARDs have a risk of COVID-19 infection, they are not at an increased risk of death.^9^

It is commonly shown that viral infections are associated with the exacerbation of IRD or *vice versa*.^10^ With respect to COVID-19, adequate and timely management of the infection in patients with IRD has generally reduced the excess mortality.^5^ However, sub-Saharan Africa presents unique challenges that may significantly alter COVID-19 outcomes in IRD patients, including limited access to specialised rheumatology care, inconsistent availability of DMARDs and biologics, different patterns of comorbidities and distinct socioeconomic factors affecting healthcare-seeking behaviour.

Specifically, no prospective studies have examined COVID-19 incidence and risk factors among IRD patients in Kenya. This evidence gap is particularly concerning given that the conflicting findings reported in global literature may not be generalisable to the Kenyan context, where treatment patterns, healthcare access and population characteristics differ substantially from high-resource settings. Furthermore, existing Kenyan COVID-19 surveillance data lack disaggregation by rheumatic disease status, making it impossible to inform evidence-based clinical guidelines for this vulnerable population.

Therefore, we conducted a prospective register-based follow-up study to evaluate the association between IRDs and COVID-19 among general patients after hospital discharge in Nairobi, Kenya, to provide the first local evidence base for optimising care strategies in this population.

## Research methods and design

### Data gathering

In this prospective cohort study, data were gathered from Mbagathi County Hospital, one of the most frequented public hospitals in the Nairobi metropolitan area, Kenya. The local health record personnel collected individual-level data from paper-based and electronic medical records, and Kenyan National Identity Card Numbers were applied to combine the patients with the records. The classification of patients as IRD and non-IRD was based on the patients’ diagnoses during hospitalisation as captured in the registries. In this study, IRDs referred to the following diagnoses: rheumatoid arthritis, acute inflammatory arthropathy, psoriatic arthritis, ankylosing spondylitis, enteropathic arthritis, rheumatic fever (without mention of heart) and systemic sclerosis. The diagnoses were based on patient history, clinical examination, laboratory and imaging findings and the International Classification of Disease, 10th Revision (ICD-10), codes assigned by attending physicians.

Patients who were admitted to the hospital between 01 January 2022 and 30 September 2022 were deemed eligible to participate in the study baseline. For each study participant, up-to-date data were acquired on patient characteristics, the hospital admission date and the reason for hospital stay. Medical records also revealed one coexisting illness in addition to the underlying illness that caused the initial hospitalisation. Out of the 4420 patients included in the study baseline, 147 (3.3%) ceased during the hospitalisation. The study baseline together with sample size calculations are reported in detail by Onchong’a et al.^11,12^ Briefly, our aim was to obtain data of at least 815 non-IRD patients in a three-to-one relationship with respect to IRD patients, namely the eligible total number of non-IRD patients was three times the total number of IRD patients (Appendix 1). This aim was based on sample size calculations^13^ in which we assumed the COVID-19 prevalence of 10% in IRD patients and a 25% lower prevalence in non-IRD patients.^14^ In this prospective study, the participants were followed up from the initial hospital discharge for COVID-19 at each hospital visit until their last hospital readmission or death within 2 years from baseline.

### Outcome and covariates

The COVID-19 status at follow-up served as the outcome. In statistical analyses, it was used as a time-to-event variable, COVID-19 negative (no) vs. positive (yes) based on reverse transcription polymerase chain reaction (RT-PCR) tests together with the date when the sample for the test was taken.

As a routine practice in Kenya, age, sex, education, income, employment, physical activity, alcohol drinking and tobacco smoking are self-reported at the admission to the hospital and recorded in the patient files, whereas weight and height are measured.

We calculated the body mass index (BMI) by dividing the weight in kilograms by the square of the height in metres. For the subgroup analyses, BMI was categorised as follows: Underweight (BMI < 18.5 kg/m^2^), healthy weight (BMI 18.5 kg/m^2^ – 24.9 kg/m^2^), overweight (BMI 25.0 kg/m^2^ – 29.9 kg/m^2^) and obesity (BMI 30.0 kg/m^2^).

Education was categorised as primary, secondary or tertiary. In accordance with the Kenyan education system, the primary category refers to the lowest level of education between Grade 1 and Grade 8. The secondary category refers to the middle level of education or high school. The tertiary category represents higher education obtained after secondary education or high school.

Income was reported as low, middle or upper. The categorisation is based on the Kenya Bureau of Statistics classification in which people belonging to the low-, middle- and upper-income categories earn £23 670.00 (≤ $183.00), £23 671.00 – £119 999.00 ($184.00 – $929.00) and ≥ £120 000.00 (≥ $930.00) Kenyan shillings (KES) per month, respectively.

Employment was categorised as formal, informal or unemployed. Formal refers to having a daily job that involves a contract between the employer and the employee. Informal refers to jobs with no contract between the employer and the employee. Unemployment refers to having no job.

Regarding the physical activity level (PAL), the categorisation was based on recommendations from the nutrition and physiotherapy departments in the local health facility. Physical activity level was counted as the number of times the patient was involved in any form of voluntary physical exercise within 1 week. Less than 4 times was categorised as low, 4–7 times as moderate and more than 7 times as high PAL.

Alcohol drinking and tobacco smoking statuses were categorised as never, previous or current (an active smoker) and dichotomised for the statistical analyses as never (no) vs. ever (yes, including previous and current categories).

The COVID-19 vaccination status was categorised as not vaccinated, partially vaccinated (1 dose) and fully vaccinated (2 or more doses).

The COVID-19 status at baseline was based on RT-PCR or antigen tests.^9^

Comorbidities retrieved from medical records were categorised according to the ICD-10.

### Statistical analysis

Descriptive analyses of baseline characteristics were conducted according to the IRD status (IRD vs. no IRD). Means and standard deviations (s.d.) were reported for continuous variables (age and BMI), frequencies and proportions for categorical variables (sex, education, income, employment, physical activity, alcohol drinking, tobacco smoking, COVID-19 vaccination and COVID-19 status). The independent samples *t*-test was used to test differences in the continuous variables between the IRD and non-IRD patients. Correspondingly, the Mann-Whitney *U* and the Pearson’s Chi-squared tests were used to test differences in the categorical variables.

A hierarchical Cox proportional hazard model was applied to predict the hazard of COVID-19 in patients with IRD versus no IRD. Hazard ratio (HR) served as the effect measure, and *p* < 0.05 was considered to indicate statistical significance. In Model 1, the hazard was adjusted for age and sex and in Model 2, for all baseline characteristics presented in Table 1. The proportional hazards assumption for the Cox model was evaluated by means of Schoenfeld residuals, covariate by covariate, and no violations were detected. Subgroup analyses were executed to reveal whether covariates confound the association between COVID-19 and IRD. Subgroup analyses refer to groups formed by baseline characteristics and comorbidities, termed as covariates. Multiplicative interactions of IRD with the subgrouping covariates were tested.

**TABLE 1:** Baseline characteristics.

Characteristic	Total	No IRD	IRD	*p*
***N***	3299	2951	348	NA
**Age in years**	-	-	-	< 0.001
Mean ± s.d.	61.8 ± 10.2	61.6 ± 10.3	63.7 ± 8.8	-
**BMI in kg/m^2^**	-	-	-	< 0.001
Mean ± s.d.	25.4 ± 3.6	25.3 ± 3.7	26.3 ± 2.8	-
**Women BMI in kg/m^2^**	-	-	-	< 0.001
Mean ± s.d.	26.2 ± 3.6	26.1 ± 3.7	27.2 ± 2.9	-
**Men BMI in kg/m^2^**	-	-	-	< 0.001
Mean ± s.d.	24.6 ± 3.4	24.5 ± 3.5	25.5 ± 2.6	-
**Sex**	-	-	-	0.841
Women	-	-	-	-
*n*	1524	1365	159	-
%	46.2	46.3	45.7	-
Men	-	-	-	-
*n*	1775	1586	189	-
%	53.8	53.7	54.3	-
**Education level**	-	-	-	0.226
Primary	-	-	-	-
*n*	528	482	46	-
%	16.0	16.3	13.2	-
Secondary	-	-	-	-
*n*	1708	1517	191	-
%	51.8	51.4	54.9	-
Tertiary	-	-	-	-
*n*	1028	923	105	-
%	31.2	31.3	30.2	-
Unknown	-	-	-	-
*n*	35	29	6	-
%	1.1	1.0	1.7	-
**Income level**	-	-	-	0.979
£23 670.00 KES/month	-	-	-	-
*n*	1296	1158	138	-
%	39.3	39.2	39.7	-
£23 671.00–£119 999.00 KES per month	-	-	-	-
*n*	1923	1721	202	-
%	58.3	58.3	58.0	-
Unknown	-	-	-	-
*n*	80	72	8	-
%	2.4	2.4	2.3	-
**Employment status**	-	-	-	0.603
Unemployed	-	-	-	-
*n*	1095	975	120	-
%	33.2	33.0	34.5	-
Informal	-	-	-	-
*n*	1525	1360	165	-
%	46.2	46.1	47.4	-
Formal	-	-	-	-
*n*	646	585	61	-
%	19.6	19.8	17.5	-
Unknown	-	-	-	-
*n*	33	31	2	-
%	1.0	1.1	0.6	-
**Physical activity level**	-	-	-	0.659
Low	-	-	-	-
*n*	881	780	101	-
%	26.7	26.4	29.0	-
Moderate	-	-	-	-
*n*	2371	2129	242	-
%	71.9	72.1	69.5	-
High	-	-	-	-
*n*	4	4	0	-
%	0.1	0.1	0.0	-
Unknown	-	-	-	-
*n*	43	38	5	-
%	1.3	1.3	1.4	-
**Alcohol drinking status**	-	-	-	< 0.001
Ever-drinker	-	-	-	-
*n*	339	280	59	-
%	10.3	9.5	17.0	-
Never-drinker	-	-	-	-
*n*	2960	2671	289	-
%	89.7	90.5	83.0	-
**Tobacco smoking status**	-	-	-	0.240
Ever-smoker	-	-	-	-
*n*	365	320	45	-
%	11.1	10.8	12.9	-
Never-smoker	-	-	-	-
*n*	2934	2631	303	-
%	88.9	89.2	87.1	-
**COVID-19 vaccination status**	-	-	-	0.031
Not vaccinated	-	-	-	-
*n*	754	663	91	-
%	22.9	22.5	26.1	-
Partially vaccinated	-	-	-	-
*n*	512	447	65	-
%	15.5	15.1	18.7	-
Fully vaccinated	-	-	-	-
*n*	2033	1841	192	-
%	61.6	62.4	55.2	-
**COVID-19 status**	-	-	-	0.585
Positive	-	-	-	-
*n*	212	192	20	-
%	6.4	6.5	5.7	-
Negative	-	-	-	-
*n*	3087	2759	328	-
%	93.6	93.5	94.3	-

IRD, inflammatory rheumatic disease; s.d., standard deviation; BMI, body mass index; KES, Kenyan shilling; NA, not applicable.

### Ethical considerations

Ethical approval to conduct this study, the data gathering procedure as well as the use of the gathered data for research purposes were authorised and ethically approved by the Kenyan Ministry of Health Research Ethics Committee (County Government of Nairobi) on 22 August 2022 (MOH/P/78/OOK8191). A pseudonymised dataset was generated for research purposes.

## Results

### Baseline characteristics

The follow-up study cohort included 3299 patients, of which 348 with IRD and 2951 with no IRD. Inflammatory rheumatic disease patients were older (mean age 64 years vs. 62 years; *p* < 0.001), heavier in relation to weight (mean BMI 26.3 kg/m^2^ vs. 25.3 kg/m^2^; *p* < 0.001), more often alcohol drinkers (17.0% vs. 9.5%; *p* < 0.001) and less often vaccinated against COVID-19 (74.0% vs. 78.0%; *p* = 0.031). No statistically significant differences were detected in other baseline characteristics between IRD and non-IRD patients (Table 1).

According to ICD-10 diagnosis blocks, the most common reason other than IRD for the baseline hospitalisation was a disease of the digestive system (*n* = 1127; 34%) followed by a disease of the circulatory system (*n* = 538; 16%) and a disease of the musculoskeletal system and connective tissue, excluding IRDs (*n* = 341; 10%) (Table 2).

**TABLE 2:** COVID-19 rate during the 2-year follow-up period by the reason for baseline hospitalisation.

Comorbidity	*n*	%	COVID-19 (*n*)	Rate (%)
Inflammatory rheumatic disease	348	11.0	20	5.7
ICD-10 diagnosis A (Septicaemia, mainly)	16	0.5	< 10	N/A
ICD-10 diagnosis B (Hepatitis A, mainly)	144	4.4	13	9.0
ICD-10 diagnosis D (Lipoma)	68	2.1	< 10	N/A
ICD-10 diagnosis E (Endocrine, nutritional or metabolic)	30	0.9	< 10	N/A
ICD-10 diagnosis G (Multiple sclerosis, mainly)	86	2.6	< 10	N/A
ICD-10 diagnosis I (Circulatory system)	538	16.0	38	7.1
ICD-10 diagnosis J (Respiratory system)	198	6.0	17	8.6
ICD-10 diagnosis K (Digestive system)	1127	34.0	65	5.8
ICD-10 diagnosis L (Psoriasis or urticaria)	117	3.5	10	8.5
ICD-10 diagnosis M (Musculoskeletal system, mainly)	341	10.0	18	5.3
ICD-10 diagnoses M and K (Fibromyalgia and IBS)	19	0.6	< 10	N/A
ICD-10 diagnosis N (Genitourinary system)	124	3.8	< 10	N/A
ICD-10 diagnosis R (Functional tachycardia)	61	1.8	< 10	N/A
ICD-10 diagnosis T (Subcutaneous emphysema)	69	2.1	< 10	N/A

Note: To protect privacy, ICD-10 diagnosis blocks with less than 10 patients were omitted from the table, and those with less than 10 COVID-19 cases are not reported in detail.

ICD-10, International Classification of Diseases, 10th Revision; IBS, inflammatory bowel disease; N/A, not applicable.

The most common comorbidities were cardiovascular diseases (CVDs) both among IRD and non-IRD patients (20.0% vs. 21.0%). Up to 98.0% of non-IRD patients had at least one coexisting illness, whereas in IRD patients, the corresponding proportion was 92.0%.

### COVID-19 and inflammatory rheumatic disease

**FIGURE 1:**
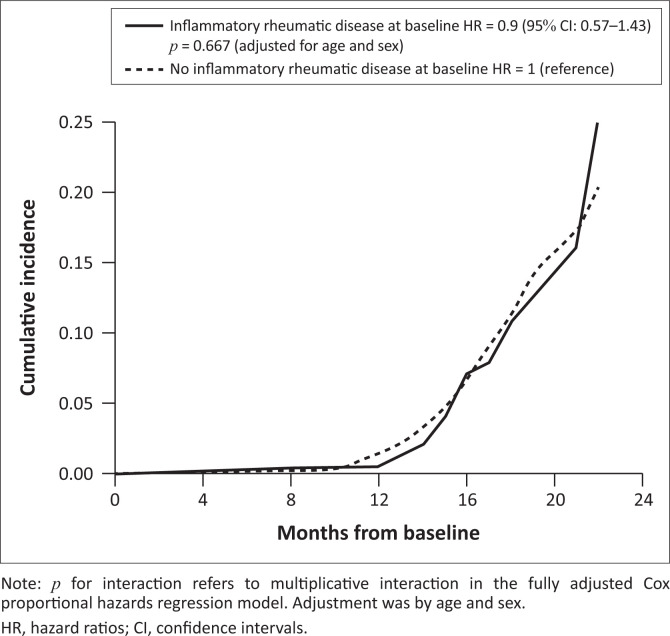
Cumulative incidence of COVID-19 by inflammatory rheumatic disease status.

Inflammatory rheumatic diseases did not increase the hazard of COVID-19 compared to non-IRD patients (HR adjusted for age and sex 0.9; 95% confidence interval [CI]: 0.6–1.4; *p* = 0.667 and fully adjusted for all baseline characteristics 0.9; 95% CI: 0.6–1.4; *p* = 0.675) (Table 3, Figure 1). The mean ± s.d. follow-up time was 15.2 ± 2.9 months, irrespective of the IRD status (yes vs. no). In IRD patients, the 2-year COVID-19 rate per 100 person-years was 4.5 (95% CI: 3–7), whereas among patients having no IRD, it was 5 (95% CI: 4–6). There was no evidence of subgroup interactions with respect to the association between COVID-19 and IRD (Table 4).

**TABLE 3:** COVID-19 rate during the 2-year follow-up period by the inflammatory rheumatic disease status.

Event	No IRD	IRD	*p*
*N*	2951	348	-
PY, *n*	3735.5	440.2	-
Events, *n*	192	20	-
Rate per 100 PY	5.14	4.54	
95% CI	4.48–5.90	2.96–6.97	-
HR†	1	0.90	-
95% CI	-	0.57–1.43	0.667

IRD, inflammatory rheumatic disease; PY, person-years; HR, hazard ratio; CI, confidence interval.

† , Adjusted for age and sex in the Cox proportional hazards regression model.

**TABLE 4:** COVID-19 rate during the 2-year follow-up period by the subgroup and the inflammatory rheumatic disease status.

Subgroup	Total		No IRD			IRD				*p*	*p*-value for interaction
	*n*	Events	*n*	Events	HR	*n*	Events	HR	CI		
**Sex**	-	-	-	-	-	-	-	-	-	-	0.654
Women	1524	108	1365	98	1	159	10	0.88	0.46-1.68	0.688	-
Men	1775	104	1586	94	1	189	10	0.92	0.48-1.77	0.802	-
**Age**	-	-	-	-	-	-	-	-	-	-	0.189
Age 24-60 years	1293	85	1194	> 10	1	99	< 10	0.59	0.22-1.62	0.307	-
Age 61-83 years	2005	127	1756	111	1	249	16	1.06	0.63-1.80	0.823	-
Age unknown	1	0	N/A	-	-	N/A	-	-	-	-	-
**BMI**	-	-	-	-	-	-	-	-	-	-	0.580
Underweight BMI <18.5 kg/m^2^	119	6	N/A		1	N/A		N/A	N/A	N/A	-
Healthy weight BMI 18.5 kg/m^2^ – 24.9 kg/m^>2^	1412	88	1299	> 10	1	113	< 10	0.70	0.28-1.73	0.441	-
Overweight BMI 25.0 kg/m^2^ – 29.9 kg/m^2^	1436	100	1234	87	1	202	13	0.93	0.52-1.67	0.806	-
Obesity BMI ≥ 30.0 kg/m^2^	332	18	301	> 10	1	31	< 10	1.06	0.24-4.71	0.944	-
**Education**	-	-	-	-	-	-	-	-	-	-	0.349
Primary education	528	39	482	> 10	1	46	< 10	0.64	0.15-2.67	0.541	-
Secondary education	1708	100	1517	> 10	1	191	< 10	0.86	0.43-1.70	0.656	-
Tertiary education	1028	72	923	> 10	1	105	< 10	1.24	0.61-2.49	0.557	-
Education unknown	35	1	N/A	-	-	N/A	-	-	-	-	-
**Income**	-	-	-	-	-	-	-	-	-	-	0.771
Income ≤ 23 670 KES/month	1296	93	1158	> 10	1	138	< 10	0.79	0.38-1.62	0.514	-
Income 23 671–119 999 KES/month	1923	114	1721	103	1	202	11	0.93	0.50-1.74	0.830	-
Income unknown	80	5	N/A	-	-	N/A	-	-	-	-	-
**Employment**	-	-	-	-	-	-	-	-	-	-	0.081
Unemployed	1095	75	975	> 10	1	120	< 10	0.45	0.16–1.23	0.119	-
Informally employed	1525	98	1360	86	1	165	12	1.16	0.63–2.12	0.635	-
Formally employed	646	-	585	> 10	1	61	< 10	1.38	0.49–3.92	0.545	-
Employment status unknown	33	2	N/A	-	-	N/A	-	-	-	-	-
**PAL**	-	-	-	-	-	-	-	-	-	-	0.383
PAL low	881	52	780	> 10	1	101	< 10	0.64	0.23–1.77	0.387	-
PAL moderate	2371	157	2129	141	1	242	16	1.03	0.62–1.74	0.899	-
PAL high	4	0	N/A	-	1	N/A	-	N/A	N/A	N/A	-
PAL unknown	43	3	N/A	-	-	N/A	-	-	-	-	-
**Smoking**	-	-	-	-	-	-	-	-	-	-	0.435
Ever-smoker	365	17	320	> 10	1	45	< 10	0.35	0.05–2.63	0.305	-
Never-smoker	2934	94	2631	175	1	303	19	0.97	0.60–1.56	0.903	-
**Drinking**	-	-	-	-	-	-	-	-	-	-	0.418
Ever-drinker	339	27	280	> 10	1	59	< 10	0.53	0.16–1.79	0.309	-
Never-drinker	2960	185	2671	168	1	289	17	0.98	0.59–1.61	0.921	-
**Vaccination**	-	-	-	-	-	-	-	-	-	-	0.960
Not vaccinated against COVID-19	754	44	663	> 10	1	91	< 10	0.81	0.32-2.07	0.661	-
Partially vaccinated against COVID-19	512	40	447	> 10	1	65	< 10	1.19	0.46–3.05	0.722	-
Fully vaccinated against COVID-19	2033	128	1841	118	1	192	10	0.85	0.44–1.62	0.615	-
**COVID-19**	-	-	-	-	-	-	-	-	-	-	0.220
No COVID-19 at baseline	2786	186	2498	172	1	288	14	0.75	0.43–1.29	0.295	-
COVID-19 at baseline	292	16	244	> 10	1	48	< 10	2.26	0.77–6.68	0.140	-
COVID-19 at baseline unknown	221	10	209	< 10	1	12	< 10	1.06	0.13–8.85	0.956	-

Note: Interactions were considered as multiplicative interactions and adjusted for age and sex. To protect privacy, subgroups with less than 10 patients and/or COVID-19 cases are not reported in detail. Hazard ratios (95% confidence intervals) were adjusted for age and sex in the Cox proportional hazards model.

IRD, inflammatory rheumatic disease; KES, Kenyan shilling; PAL, physical activity level; HR, hazard ratios; N/A, not applicable; CI, confidence interval.

Comorbidities did not affect the association between COVID-19 and IRD, but the hazard rate of COVID-19 in IRD compared to that in non-IRD patients was statistically non-significant, irrespective of the coexisting disease (Table 5). Only 76 (2.3%) patients had no comorbidities, and among them, IRD did not increase the hazard of COVID-19 compared to patients with no IRD and no coexisting disease (HR = 0.4; 95% CI: 0.1–2.2; *p* = 0.437).

**TABLE 5:** COVID-19 rate during the 2-year follow-up period by the main comorbidity and the inflammatory rheumatic disease status.

Comorbidity	Total		No IRD (HR)	IRD		*P*
	*n*	Events		HR	CI	
No comorbidities	76	10	1	0.44	0.09–2.16	0.437
ICD-10 diagnosis A	180	16	1	0.82	0.18–3.80	0.801
ICD-10 diagnosis B	235	15	1	0.50	0.07–3.90	0.510
ICD-10 diagnosis D	418	30	1	1.24	0.37–4.13	0.722
ICD-10 diagnosis E	353	26	1	1.29	0.38–4.40	0.689
ICD-10 diagnosis I	686	33	1	0.70	0.17–2.96	0.632
ICD-10 diagnosis J	204	17	1	0.91	0.12–7.07	0.926
ICD-10 diagnosis K	166	10	1	0.77	0.09–6.55	0.811
ICD-10 diagnosis N	239	21	1	0.89	0.25–3.08	0.847
ICD-10 diagnosis R	410	25	1	0.34	0.05–2.51	0.289

Note: To protect privacy, ICD-10 diagnosis blocks with less than 10 COVID-19 cases were omitted. Hazard ratios (95% confidence intervals) were adjusted for age and sex in the Cox proportional hazards model.

IRD, inflammatory rheumatic disease; HR, hazard ratios; CI, confidence intervals; ICD-10, International Classification of Diseases, 10th Revision.

The crude COVID-19 rate over the 2-year follow-up was highest in patients with ICD-10 category B diagnoses, mainly hepatitis A (9.0%) followed by patients with respiratory system diseases (8.6%) and patients with psoriasis or urticaria (8.5%) (Table 2).

## Discussion

### Main findings

This longitudinal study of 3299 patients showed that the presence of IRD did not significantly increase the hazard of COVID-19 when adjusted for age, sex and BMI. The 2-year COVID-19 rate was nearly equal in IRD and non-IRD patients. There were no significant subgroup interactions between the IRD status (no vs. yes) and potential confounders with respect to COVID-19. The presence of comorbidities did not increase the IRD patient’s risk of COVID-19 within the 2-year follow-up. The highest crude COVID-19 rates were observed in patients with hepatitis A, respiratory diseases or psoriasis.

### Comparison with previous studies and possible explanations for findings

While other studies have reported an increased risk of COVID-19 infection among IRD patients,^15^ others have reported that IRD might not, independently and substantially, impact the risk of COVID-19.^6,16,17,18,19^ The present prospective study of an initially inpatient cohort from the Nairobi metropolitan area supports the latter findings.

A large-scale study involving 66 840 patients with IRD in Denmark found that while IRD patients had a slightly higher incidence of COVID-19 infection compared to the general population, their overall risk was not markedly elevated.^16^ The study emphasised that the increased severity of COVID-19 outcomes in IRD patients was more closely linked to the presence of comorbidities rather than IRD itself.^16^ Another study analysing COVID-19 in French patients with chronic IRDs did not observe any increase in the incidence or severity of COVID-19 in patients suffering from spondylarthritis, rheumatoid arthritis or psoriatic arthritis.^17^ The same study emphasised that the treatment with immunosuppressive therapies did not cause an increased risk of COVID-19 infection.^17^ Sachdeva et al. reported a low COVID-19 prevalence in rheumatic disease patients.^13^ In addition, they found out that COVID-19 plays no role in determining disease severity and outcomes.^18^ These findings could be because of the IRD patients being aware of the autoimmune nature of their disease and the associated immunosuppressive drug therapy might be more vigilant in healthcare seeking in case of early respiratory symptoms. In addition, treatment with biologic synthetic DMARDs has been associated with a protective role against COVID-19 infection.^19^ A decreased risk of COVID-19-related hospital admission in newly diagnosed IRD patients was reported in patients using biologic synthetic DMARDs as compared to their peers with no DMARDs.^6^ Another reason for a non-association between COVID-19 and IRD could be because of missed diagnoses, especially during flare-ups of rheumatic diseases in which symptoms such as fatigue, increased erythrocyte sedimentation and laboratory abnormalities might mimic those observed in COVID-19.^18^

Interestingly, having a coexisting disease was not associated with an increased risk of COVID-19 in the present study. This is not in accordance with previous studies that have reported an increased risk of COVID-19 when IRD coexists, for example, with CVD and diabetes mellitus.^20,21,22^ Our findings concur with results of a Brazilian study based on which the comorbidity burden in patients with rheumatoid arthritis and COVID-19 does not differ from that in the general population.^23^ Yet another study found no significantly increased risk for COVID-19 hospitalisation among IRD patients regardless of their IRD diagnoses.^24^ According to a meta-analysis, there is no clear general evidence that the risk of COVID-19 infection would increase together with the number of comorbidities.^25^

Our finding that the COVID-19 incidence was relatively high among patients with respiratory diseases is consistent with previous studies.^26,27,28^ A possible explanation for the association between respiratory diseases and the elevated susceptibility to COVID-19 may arise from the occurrence of prolonged immunological dysfunctions caused by COVID-19. This leads to increased expression of interferons and other proinflammatory cytokines associated with chronic respiratory diseases.^23^

### Clinical and public health implications

Our findings have important implications for clinical practice in managing IRD patients during ongoing and future pandemic situations. The lack of significantly increased COVID-19 risk among IRD patients suggests that clinicians should not automatically consider IRD status alone as a major risk factor for COVID-19 infection when making treatment decisions or risk stratification. This finding supports continuing standard IRD treatments, including immunosuppressive therapies and biologic DMARDs, without necessarily modifying regimens solely based on COVID-19 concerns.

However, the elevated COVID-19 rates observed in patients with specific conditions such as hepatitis A, respiratory diseases and psoriasis indicate that clinicians should focus heightened surveillance and preventive measures on these particular subgroups rather than broadly categorising all IRD patients as high risk. The protective effects suggested for biologic synthetic DMARDs also support maintaining these treatments in appropriate patients, as discontinuation may cause more harm through disease flares than continued use during viral outbreaks.

From a public health perspective, our findings suggest that pandemic preparedness strategies should avoid general categorisation of all IRD patients as a uniformly high-risk population for COVID-19. Instead, public health interventions should be more targeted on IRD patients with specific high-risk comorbidities rather than implementing broad restrictions based solely on IRD diagnosis. The findings also support the importance of maintaining routine rheumatology care during pandemics, as the benefits of continued treatment appear to outweigh COVID-19-related risks for most IRD patients. For health systems in similar resource-limited settings such as Kenya, these results provide locally relevant evidence to guide policy decisions about IRD patient management during health emergencies, moving away from precautionary approaches based primarily on international data from different healthcare contexts.

### Strengths and limitations

To the best of our knowledge, this is the first longitudinal study in the geographical region of East Africa addressing the association between COVID-19 and IRDs. The use of a longitudinal design made it possible to establish overtime associations between IRD status and COVID-19. Moreover, this study retrieved follow-up data covering a wide range of variables (demographic, socioeconomic, lifestyle factors and comorbidities) allowing analyses controlled for potential confounders.

As a potential limitation, data were collected from only one public hospital in Nairobi, which may limit the generalisability of the findings to other regions in Kenya or to different types of healthcare settings. The lack of extensive population testing facilities for COVID-19 in Kenya, in general, might have led to underreporting of cases among patients with IRD and other underlying conditions, leading to an underestimation of the true risk also in the present study. In a Kenyan survey carried out in August 2021, approximately 45% out of 419 study participants wished to be tested for COVID-19, but they were unable to access the testing service because of inadequate laboratory diagnostic services across the country.^29^ In addition, our study had the mean follow-up time of only 15 months, which may not be capable of capturing long-term effects of IRD on COVID-19, including its delayed outcomes. As a result, we suggest to cautiously generalise the present results and to use them as a reference for the future IRD research, particularly in the sub-Saharan African region.

## Conclusion

Inflammatory rheumatic diseases were not associated with an increased hazard of COVID-19 in the present Nairobian cohort within the mean follow-up of 15 months. The collection of demographic, socioeconomic, lifestyle and comorbidity characteristics of study participants we were able to take into account in this study did not alter the association between COVID-19 and IRD, but it remained statistically non-significant. These findings suggest that IRD does not pose a great risk for the COVID-19 infection in the Nairobi metropolitan area, not even in the presence of other major health issues, such as comorbidities, obesity and tobacco smoking. The crude incidence rate of COVID-19 was highest among patients with hepatitis A, respiratory diseases or psoriasis.

## Appendix 1

*Sample size calculations*. We applied the equations from Rosner^1^ to calculate the required sample size as follows:

**Table:**

*p*_*1*_	Expected COVID-19 prevalence in IRD patients	10/100 = 0.1
*q*_*1*_	1–*p*_1_	0.9
*p*_*2*_	Expected COVID-19 prevalence in non-IRD patients	(10 × 0.75)/100 = 0.075
*q*_*2*_	1–*q*_1_	0.925
*∆*	|*p*_2_ – *p*_1_|	0.025
*k*	The expected ratio of non-IRD (*n*_1_) to IRD patients (*n*_2_)	3
$\bar{p}$	(*p*_1_ + *kp*_2_) / (1 + *k*)	0.08125
$\bar{q}$	1– $\bar{p}$	0.91875
α	Significance level	0.05
1–β	Power	0.8
*z*_1–α/2_	Respective *z*-score of the standard normal distribution	1.96
*z*_1–β_	Respective *z*-score of the standard normal distribution	0.84

$$\begin{array}{l} n_{1}=\frac{{\left[\sqrt{\bar{pq}\left(1+\frac{1}{k}\right)}z_{1-\alpha /2}+\sqrt{p_{1}q_{1}+\frac{p_{2}q_{2}}{k}}z_{1-\beta }\right]}^{2}}{\Delta^{2}}\\ n_{2}=kn_{1}\\ n_{1}=\frac{{\left[\sqrt{0.074648\times 1.333333}\times 1.96+\sqrt{0.09+0.023125}\times 0.84\right]}^{2}}{0.000625}=814.2509\\ n_{2}=kn_{1}=2442.753 \end{array}$$
